# The complete mitochondrial genome of the seed-borer weevil, *Bruchidius uberatus* (Coleoptera: Chrysomelidae: Bruchinae)

**DOI:** 10.1080/23802359.2019.1698331

**Published:** 2019-12-13

**Authors:** Li-Jie Zhang, Ling Wu, You Li, Jian-Guang Li, Xing-Ke Yang, Rui-E Nie

**Affiliations:** a Beijing Customs District P. R. China, Beijing, China;; b Key Laboratory of Zoological Systematics and Evolution, Institute of Zoology, Chinese Academy of Sciences, Beijing, China;; c School of Forest Resources and Conservation, University of Florida, Gainesville, FL, USA

**Keywords:** Mitochondrial genome, phylogeny, *Bruchidius uberatus*, Bruchinae

## Abstract

In this study, the complete 15,892 bp mitochondrial genome of *Bruchidius uberatus* (Fåhraeus) was sequenced using Illumina NovaSeq6000 platform. The mitogenome is a double-stranded circular molecule of 15,892 bp in length with 22 transfer RNA genes, 13 protein-coding genes and two ribosomal RNA genes as in other insects. Twenty-five species from 8 subfamilies of Chrysomelidae were selected as ingroups and 3 species of Lamiinae as outgroups for phylogenetic analysis based on mitogenome. The results showed that the subfamily Bruchinae was monophyly. Genus *Bruchidius* had more closed relationship with *Acanthoscelides* than *Callosobruchus* in Bruchinae with high support values.

*Bruchidius uberatus* (Fahraeus) (Coleoptera: Chrysomelidae: Bruchinae) is a serious pest of *Vachellia nilotica* seeds (including *V. n. tomentosa* and *V. n. adansonii*), a far-spread tree species in African savannas with important economic and ecological value. In addition to the main host plant, this bruchid is also listed from *Senegalia senegal, Vachellia sieberiana*, *V. flava, V. tortilis, V. kirkii, V. seyal* and *Acacia ehrenbergiana* (Ernst et al. 1990; Delobel et al. 2015).

The specimens used in this study were intercepted in imported *Vachellia* sp. from Nigeria (N9°3′14.24″E7°29′17.57″). The sequenced DNA was kept at the National Zoological Museum of China, Institute of Zoology, Chinese Academy of Sciences, Beijing, China (NZMC, the DNA accession number: DX104). The species was identified by Dr. You Li. The complete mitogenome of *B. uberatus* was sequenced by Illumina’s HiSeq6000 platform (Illumina, San Diego, CA, USA) with 350 bp insert size and a pair-end 150 bp sequencing strategy. The sequence reads were first filtered by the programs following Zhou et al. (2013) and then the remaining high-quality reads were assembled using IDBA-UD (Peng et al. 2012). The annotations of genes were done by Geneious 8.0.5 software (Kearse et al. 2012) and tRNAscan-SE 1.21 (Schattner et al. 2005).

The complete mitochondrial genome (mitogenome) of *B. uberatus* is a double-stranded circular molecule of 15,892 bp in length (GenBank accession number: MN594498), with 22 transfer RNA genes, 13 protein-coding genes and two ribosomal RNA genes as in other insects. The overall base composition is A: 39.3%, T: 38.2%, C: 13.4%, and G: 9.1%, with a much higher A + T content.

For the phylogenetic analysis, all available mitogenomes of the subfamilies of Chrysomelidae was downloaded and analyzed. The acceptable sequences including 13 protein-coding genes and longer than 10 K bp were kept. Total twenty-five species (accession numbers: KY856743, KY856744, KY856745, KY942060, KY942061, KY942062, MF960125, JX412832, MF925724, MN594498, AF467886, JX412769, KF669870, KF658070, MF946616, MF960113, MF960117, MF960109, NC_028332, JX220992.1, HQ232809, JX412804, JX412756, JX220988, JX412753) from 8 subfamilies (Bruchinae, Criocerine, Cassidinae, Eumolpinae, Cryptocephalinae, Chrysomelinae, Galerucinae, Alticnae) were selected as ingroups and 3 species of Lamiinae (accession numbers: DQ768215, NC_022671, FJ424074) were selected as outgroups. The phylogenetic inference was based on 13 Protein coding genes (PCGs). TransAlign methods were used to align all genes (Bininda-Emonds 2005). The aligned data from 13PCGs were concatenated with Sequence Matrix v.1.7.8 (Vaidya et al. 2011). Bayesian inference was performed using MrBayes v.3.2 (Ronquist et al. 2012). Data were partitioned according to loci of 13 PCGs. The MCMC search was conducted for 1,000,000 generations, and sampling was done every 100 generations until the average standard deviation of split frequencies was below 0.01. The first 25% of trees were discarded as ‘burn-in’ and posterior probabilities were estimated for each node.

Phylogenetic analyses (Figure 1) showed that the subfamily Bruchinae was monophyly. Genus *Bruchidius* had more closed relationship with *Acanthoscelides* than *Callosobruchus* in Bruchinae with high support values. The position of Bruchinae in Chrysomelidae was not stable by the results of Nie et al. (2019). In this study, ‘chrysomeline’ clade (Chrysomelinae, Galerucinae, Alticnae) formed a well supported basal branch, the ‘sagrine’ clade (Criocerine) and ‘eumolpine’ clade (Cassidinae, Eumolpinae, Cryptocephalinae) formed another branch, which was close to Bruchinae. Bruchinae was not nested into ‘sagrine’ clade, may because the sampling of ‘sagrine’ clade is limited and the data type and tree building methods are different. More thorough taxon sampling and diversiform tree building methods will be needed to well understand the status of Bruchinae in Chrysomelidae.

**Figure 1.**
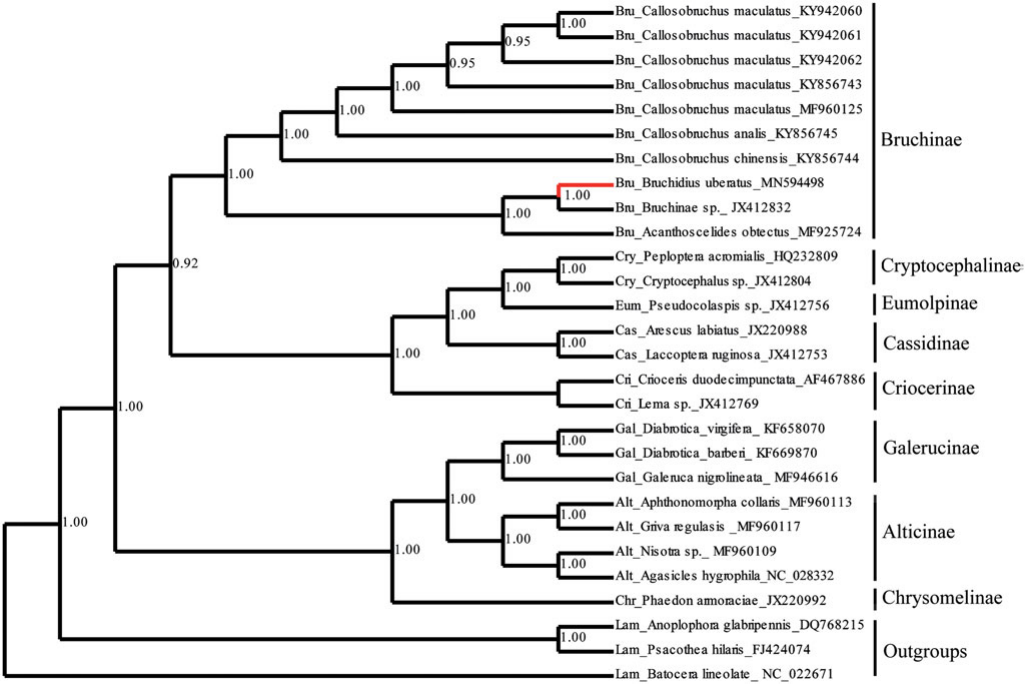
The Bayesian tree based on 13 PCGs combined data sets. Numbers on nodes indicate Bayesian posterior probabilities. Grey branch is the new data in this study.
